# MRI Study of the Influence of Surface Coating Aging on the In Vivo Biodistribution of Iron Oxide Nanoparticles

**DOI:** 10.3390/bios8040127

**Published:** 2018-12-12

**Authors:** Susana Carregal-Romero, Sandra Plaza-García, Rafael Piñol, José L. Murillo, Jesús Ruiz-Cabello, Daniel Padro, Angel Millán, Pedro Ramos-Cabrer

**Affiliations:** 1 Molecular and Functional Biomarkers group, CIC biomaGUNE, 20014 Donostia-San Sebastián, Spain; jruizcabello@cicbiomagune.es; 2 CIBER de Enfermedades Respiratorias (CIBERES), Madrid, Spain; 3 Magnetic Resonance Imaging Laboratory, CIC biomaGUNE, 20014 Donostia-San Sebastián, Spain; splaza@cicbiomagune.es (S.P.-G.); dpadro@cicbiomagune.es (D.P.); 4 CSIC-Universidad de Zaragoza, ICMA, 50009 Zaragoza, Spain; pinol@unizar.es (R.P.); jlmpola@unizar.es (J.L.M.); amillan@unizar.es (A.M.); 5 Ikerbasque, Basque Foundation for Science, 48013 Bilbao, Spain; 6 Departamento de Química en Ciencias Farmacéuticas, Universidad Complutense de Madrid, 28040 Madrid, Spain

**Keywords:** iron oxide nanoparticles, multimodal nanoparticles, biodistribution, magnetic resonance imaging, aging, coating degradation

## Abstract

Medical imaging is an active field of research that fosters the necessity for novel multimodal imaging probes. In this line, nanoparticle-based contrast agents are of special interest, since those can host functional entities either within their interior, reducing potential toxic effects of the imaging tracers, or on their surface, providing high payloads of probes, due to their large surface-to-volume ratio. The long-term stability of the particles in solution is an aspect usually under-tackled during probe design in research laboratories, since their performance is generally tested briefly after synthesis. This may jeopardize a later translation into practical medical devices, due to stability reasons. To dig into the effects of nanoparticle aging in solution, with respect to their behavior in vivo, iron oxide stealth nanoparticles were used at two stages (3 weeks vs. 9 months in solution), analyzing their biodistribution in mice. Both sets of nanoprobes showed similar sizes, zeta potentials, and morphology, as observed by dynamic light scattering (DLS) and transmission electronic microscopy (TEM), but fresh nanoparticles accumulated in the kidneys after systemic administration, while aged ones accumulated in liver and spleen, confirming an enormous effect of particle aging on their in vivo behavior, despite barely noticeable changes perceived on a simple inspection of their structural integrity.

## 1. Introduction

Medical imaging is seeking to overcome inherent limitations regarding sensitivity, specificity, resolution, and scanning time, by means of acquisition of multimodal images. This has motivated the development of novel scanners able to combine several imaging techniques (e.g., positron emission tomography and magnetic resonance imaging PET/MRI scanners), and the growth of a research field focused on the fabrication of multimodal imaging probes (contrast agents). In this line, Magnetic Resonance Imaging (MRI) is a non-invasive imaging technique with superb capacity to image soft tissues at high resolution and contrast, regardless of the depth within the body. Because of its inherent relatively low sensitivity, in comparison with other imaging techniques (e.g., nuclear imaging), and to speed up acquisition times, an important research activity exists around MRI, aiming at producing MRI contrast agents. Those agents usually combine their magnetic properties with other physical properties (e.g., fluorescence or radioactive decay) for multimodal imaging purposes [1,2,3,4]. In this context, iron oxide nanoparticle (IONP)-based contrast agents seem ideal [5,6], first, because of their already proven effectiveness as MRI contrast agents [7,8]. Indeed, the use of iron oxide nanoparticles (IONPs) as MRI contrast agents started 20 years ago, becoming very popular due to their ability to dramatically reduce T2 relaxation times in organs such as liver, spleen, and bone marrow, by selective uptake and accumulation in cells belonging to the mononuclear phagocyte or reticuloendothelial system (RES). In second place, because nanoparticles have the capacity of being labeled with other imaging probes, both on their surface, via chemical bonding or chelation within a coordination complex, and in their interior, by doping the crystalline structure [9,10,11,12]. The fabrication of multimodal nanoparticles based on the chemical functionalization of their organic coating is nowadays skeptically accepted, because several studies have shown the loss of such coatings in organic fluids, or by enzymatic degradation in vivo [13,14,15]. Therefore, the alternative doping of iron oxide nanocrystals with other probes, such as ^111^In or ^64^Cu, is gaining more interest, and the number of novel synthetic protocols has risen in the last years [3,10,16,17]. In this way, the integrity of the nanoparticle and the multimodal imaging function can be secured.

As important as keeping the integrity of the nanoparticles is the prediction and control of their fate for the effective application in nanomedicine. The choice of the administration route is one of the major factors that affects biodistribution. Intravenous injection (IV) is the most commonly used route for IONPs in MRI. Unfortunately, most of “naked” IONPs intravenously injected are rapidly filtered out by the mononuclear phagocyte system (MPS) also known as reticuloendothelial system (RES), reducing their efficacy for diagnosis [18]. The RES formed by monocytes and macrophages eliminates pathogens or foreign bodies, such as IONPs, by phagocytosis. The elimination process involves three steps: (a) Opsonization, where plasma proteins deposit on the surface of the IONPs, (b) recognition by macrophages, and (c) phagocytosis, which consists of the engulfment of the IONPs for subsequent degradation and metabolism. Therefore, liver and spleen are the major clearance pathways for the IONPs from blood, unless high doses are injected to cause the saturation of these organs and the excess IONPs accumulate in other parts of the RES [19]. Fortunately, IONPs can be tailored (i.e., we can modulate surface coating, shape, etc.) to increase their blood circulation times by making them unrecognizable to the RES system [20,21,22].

Nanoparticle size is one of the most important factors determining the biodistribution routes and kinetics. IONPs with a diameter larger than 100 nm tend to accumulate in liver and spleen, while nanoparticles smaller than 10–15 nm are normally eliminated by renal clearance [21,23,24]. Nanoparticles with a diameter between 10 and 100 nm have, in principle, longer blood circulation times, making possible the access to other tissues or organs [25,26]. In order to ensure an uniform biodistribution, IONPs should have low polydispersity index (PDI), but more importantly, size stability is highly required to avoid any type of aggregation [27,28], which is sometimes caused by inappropriate surface coating [29]. Nanoparticle aggregates are quickly trapped by the RES. [30] Among the different types of coatings, polyethylene glycol (PEG) is the most widely used, providing stability to the IONPs via steric hindrance, as well as excellent anti-fouling properties, decreasing blood and serum protein interactions, which subsequently minimizes opsonization, macrophage uptake, and RES clearance, increasing the blood circulation time of the IONPs [31,32].

Here, we report the influence of the long term stability of PEG coating on the biodistribution of grape-shaped iron oxide nanoparticles, designed as dual contrast agents for MRI and single photon emission computerized tomography (SPECT) imaging. We have recently reported the synthesis of these particles by the co-precipitation of ^111^In-doped magnetic nanoparticles, and their further coating with polyethylene glycol [17]. We describe the effect of aging of those nanoprobes in solution, relating the physicochemical modifications suffered by the particle with the changes in their in vivo biodistribution in mice, as determined by MRI. A set of freshly prepared nanoparticles (3 weeks in solution) was compared to an aged batch (circa 9 months in solution). Both sets of particles showed similar size distributions, zeta potentials and morphology, as observed by DLS and TEM, but after systemic administration in mice, the freshly prepared particles accumulated mostly in the kidneys, while the aged ones mostly accumulated in liver. Our results demonstrate how even small changes at the structural level may have a radical influence on the in vivo behavior of nanomaterials.

## 2. Materials and Methods

### 2.1. Synthesis of Grape Shaped IONPs

The preparation of IONPs and further PEG coating was carried out by a first step of formation of poly(4-vinyl pyridine) (P4VP) coated maghemite via co-precipitation, and a final PEG coating via the formation of pyridine-acrylate bonds through a Michael reaction, with acrylated PEG ligands. The grape shape is obtained during the PEG coating step. In this work, ^111^In was not included in the synthesis, as previously described [17], since only the MRI imaging modality was used to analyze the biodistribution of the nanomaterials. The different batches were prepared following exactly the same protocol, the only difference between them being the post-synthesis aging in solution (3 weeks vs. circa 9 months). Further detailed information about the synthesis procedure is provided in previous work [17].

### 2.2. Sample Characterization

The concentration of iron in each sample has been determined by Inductively Coupled Plasma-Optical Emission Spectroscopy (ICP-OES Thermo Elemental IRIS Intrepid). A LaB6-TEM, type JEOL JEM-1400PLUS (40 kV–120 kV) equipped with a GATAN US1000 CCD camera (2 K × 2 K, Gatan, Pleasanton, CA, USA) was used to image iron oxide nanoparticles. A ζ-Sizer Malvern Instrument was used for the Dynamic Light Scattering measurements. All studies were performed in backscattering mode at a 173° scattering angle, with temperature controlled at 25 °C.

### 2.3. Relaxometry Measurements

In vitro characterization of MR properties of the IONPs was performed prior to in vivo studies, determining their longitudinal and transverse relaxivities (r1 and r2, respectively). These measurements were carried out at 37 °C on a Bruker Minispec MQ60 instrument (Bruker Biospin GmbH, Ettlingen, Germany). All experiments were performed using a total volume of 300 µL of sample (ca. 0.12 mM iron concentration) to prepare a series of dilutions, using HPLC grade water as solvent. Longitudinal (T1) and transverse (T2) relaxation times were determined using the inversion-recovery and the CPMG (Carr-Purcell-Meiboom-Gill) methods, respectively. T1 and T2 relaxation times allowed us to determine the corresponding relaxivities (r1 and r2) fitting the relaxation rate (R1 and R2) dependence of the concentration using the following Equation:

R1,2 = (1/T1,2) = R1,2_0_ + r1,2 [CA], (1)

where R1_0_, R2_0_, R1, and R2 are the relaxation rates before and after contrast agent, [CA] is the concentration of the contrast agent, and r1 and r2 are the magnetic relaxivities. 

### 2.4. Magnetic Resonance Imaging (MRI)

MRI in vivo studies were performed using a 7 T horizontal bore Bruker Biospec USR 70/30 MRI system (Bruker Biospin GmbH, Ettlingen, Germany), interfaced to an AVANCE III console, and with a BGA12-S imaging gradient insert (maximal gradient strength 400 mT/m, switchable within 80 µs).

Animal experiments were conducted in our institutional animal facility, which holds a full accreditation from the Association for the Assessment and Accreditation of Laboratory Animal Care International (AAALAC). All animal procedures were approved by our Institutional animal care and use committee (IACUC), and local authorities (Diputación Foral de Guipuzcoa, Spain).

For the in vivo studies, a group of n = 6 and a group of n = 7 eight-weeks old BALB/cJRj female mice (21.2 ± 2 g body weight; Janvier, France) were used to test the biodistribution of the aged and fresh IONPs, respectively.

Imaging was achieved with a 40 mm inner diameter volume-coil working in quadrature for both signal transmission and reception. Animal preparation was started by inducing anesthesia with 5% isoflurano (reduced to 2–3% during image acquisition), in a 35/65% mixture of O_2_/N_2_ a carrier gas. The tail vein was catheterized (Polyethylene catheter attached to a 30 G needle) for the posterior intravenous injection of the contrast agent inside of the magnet. Animals were prevented from hypothermia with the use of a water blanket, maintaining the temperature at 37 ± 1 °C. To ensure animal welfare, temperature and respiration rate were continuously monitored while they remain in the MRI magnet, using a SAII M1030 system (Small animal instruments, Stony Brook, NY, USA), also used to synchronize image acquisition with the respiration of the animal. MRI scanning started with baseline scans prior to the injection of the IONPs. Then, 100 µL of contrast agent (200 µg/mL of Fe in NaCl 0.007 M and pH 7.5) was injected to the animals inside the magnet, acquiring MR images up to 120 min post injection, in intervals of 20 min. Anatomical T1-weighted axial and coronal images were acquired pre and post-injection of the contrast agent with a FLASH (Fast Low Angle Shot) sequence (TE = 4 ms; respiration synchronized (TR = 600 ms); FA = 30°; NA = 2; Matrix = 256 × 256 points; FOV = 40 × 40 mm; spatial resolution = 156 × 156 um; 20–24 slices of 1 mm thickness covering the major extension of the organs of interest at abdominal level). For quantification, T2 maps were acquired using a MSME (multi-slice multi-echo) pulse sequence (20 echoes equally spaced, ranging TE = 8–160 ms with ΔTE = 8 ms); TR = 3 × respiration rate (~60 breaths per min giving a TR~3 s); NA = 2; Matrix = 160 × 160 points; FOV = 24 × 24 mm; spatial resolution = 150 × 150 um; 8–12 slices of 1 mm thickness in 2 slice packages, one covering the kidneys/spleen region and the other the liver region).

### 2.5. In Vivo MRI Quantification

T2 parametric maps were generated on a pixel-by-pixel basis fitting the acquired images to a 3 parameter exponential decay using the Levenberg-Margardt method:

S = C+ S_0_ exp (−TE/T2), (2)

where S_0_ represent the signal intensity (S) at echo time TE = 0 ms, C is an offset, and TE is the independent variable (echo time). All image fittings have been performed with customized routines for the NIH image-J software. Mean differences in T2 relaxation times (ΔT2 = T2_t_ − T2_pre-contrast_) were calculated for each time-point (t), in manually selected regions of interest (ROIs: liver, spleen, kidney cortex, and kidney medulla). For the analysis, all the slices where the organ of interest was visible were included, giving a representative bulk value for T2 for each of the analyzed organs. It should be emphasized that pixels in these regions presenting large T2 values, corresponding to fluids such as blood or bile (gallbladder and bile ducts in the liver, and large blood vessels in the liver and kidneys) were excluded from the quantitative analysis. Thus, a maximal cutoff threshold level for T2 was set to 30, 20, or 50 ms for the liver, spleen, and kidneys, respectively. These values are beyond a safe limit of mean + 2 SD (standard deviation of the mean) for each organ.

## 3. Results and Discussion

### 3.1. Influence of Aging on the Physicochemical Properties of the Grape Shaped IONPs

**Figure 1:**
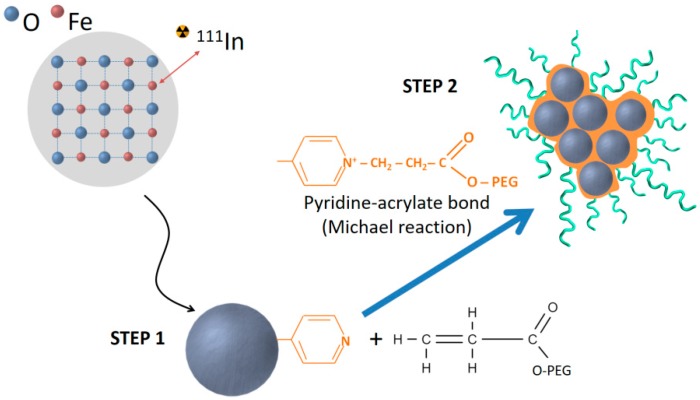
Schematic representation of the synthetic protocol of the iron oxide nanoparticles (IONPs). During STEP 1, small single IONPs are formed by coprecipitation and coated with P4PV. STEP 2 corresponds to the surface functionalization with acrylate terminated polyethylene glycol (PEG) through a Michael reaction with P4PV.

Grape-shaped iron oxide nanoparticles were produced as described elsewhere [17]. Figure 1 shows a schematic representation of the two different steps of the synthesis protocol. First, single and small iron oxide nanoparticles with an average diameter of 3.8 nm were produced, followed by polyethylene glycol coating by pyridine acrylate bonding to the surface of the nanoparticles.

**Figure 2:**
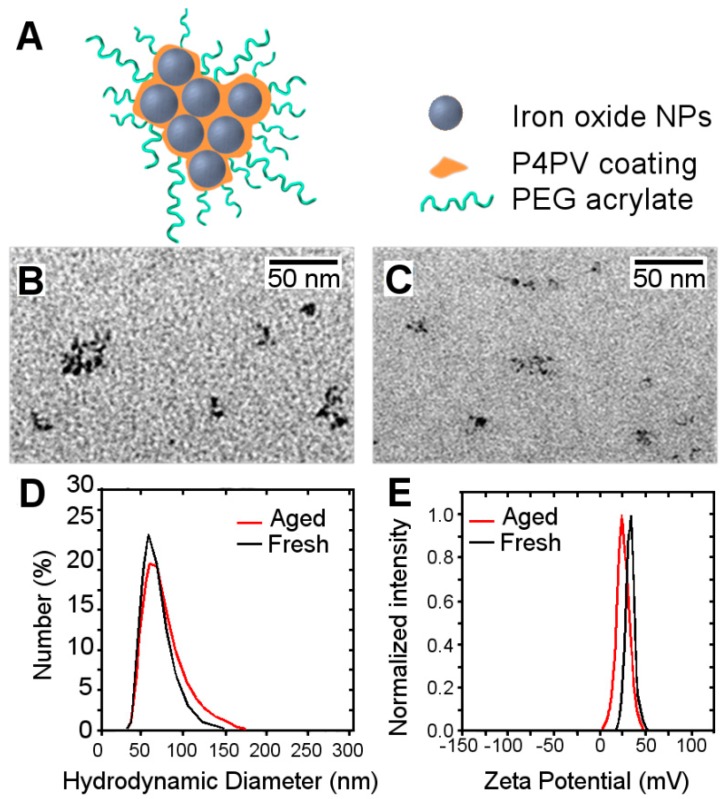
(**A**) Schematic representation of IONPs and their components. Transmission electronic microscopy (TEM) images of freshly prepared IONPs (**B**) compared with aged IONPs (**C**). Hydrodynamic diameter ((**D**), in nm) of fresh (black line) vs. aged (grey line) solutions. (**E**) Zeta potential (in mV) of fresh (black line) and aged (grey line) IONPs’ solutions.

IONPs were characterized by transmission electron microscopy after different times in solution, following synthesis. Freshly prepared solutions were compared to aged ones. Figure 2B,C show TEM images of the 2 sets of nanoparticles, finding no significant differences between them, with similar sizes and degree of agglutination observed for both sets. DLS was also used to determine the hydrodynamic diameter of the samples (Figure 2D), showing no significant differences between fresh (52.1 nm) and aged solutions (55.9 nm). Measurement of the zeta potentials also resulted in no significant differences (26.0 ± 0.4 vs. 24.1 ± 0.3 mV) between the two sets of nanoparticles, demonstrating that the charge of the nanoparticles was not significantly influenced by the time that nanoparticles remained in solution.

### 3.2. MR Relaxometry

**Figure 3:**
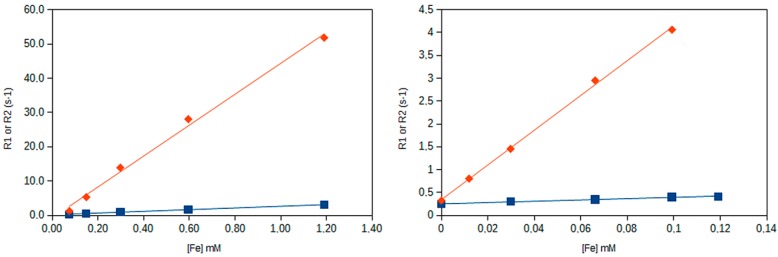
Variation of the longitudinal (R1, blue squares) and transverse (R2, red diamonds) relaxation rates (the reciprocal of T1 and T2 relaxation times) with iron concentration (as determined by inductively coupled plasma-optical emission spectroscopy (ICP-OES)) for the aged left, and freshly prepared (right) solutions of nanoparticles, at 1.41 T.

Relaxometry measurements of the IONPs for different dilutions allowed us to represent the dependence of the relaxation rates (R1 and R2) with iron concentration (Figure 3).

The corresponding relaxivities found for the contrast agent where r1 = 1.42 mM (Fe)^−1^s^−1^ and r2 = 38.0 mM (Fe)^−1^s^−1^ for the freshly prepared solution and r1 = 2.5 mM (Fe)^−1^s^−1^ and r2 = 45 mM (Fe)^−1^s^−1^ for the aged solutions (r2/r1 ratios of 26.8 and 18.4, respectively). These differences are not considered significant.

MRI contrast agents are usually classified as positive (T1) or negative (T2) depending on the value of the r2/r1 ratio [5,11]. While T1 contrast agents present a high r1 value and low (<5) r2/r1 ratio [5,11], T2 contrast agents exhibit the opposite behavior, with a high r2 value and a high (>10) r2/r1 ratio. Thus, this nanomaterial can be classified as a typical T2 contrast agent, as expected. Transverse relaxivities obtained for these IONPs were comparable to those that other authors have reported for similar ultra-small magnetic colloids [5].

### 3.3. In Vivo MRI Results

Following the characterization of IONPs, in vivo biodistribution was studied in mice with either fresh or aged IONPs solutions. As it has been already described, size, shape, and physicochemical properties of both solutions presented no significant differences, at the moment of use.

**Figure 4:**
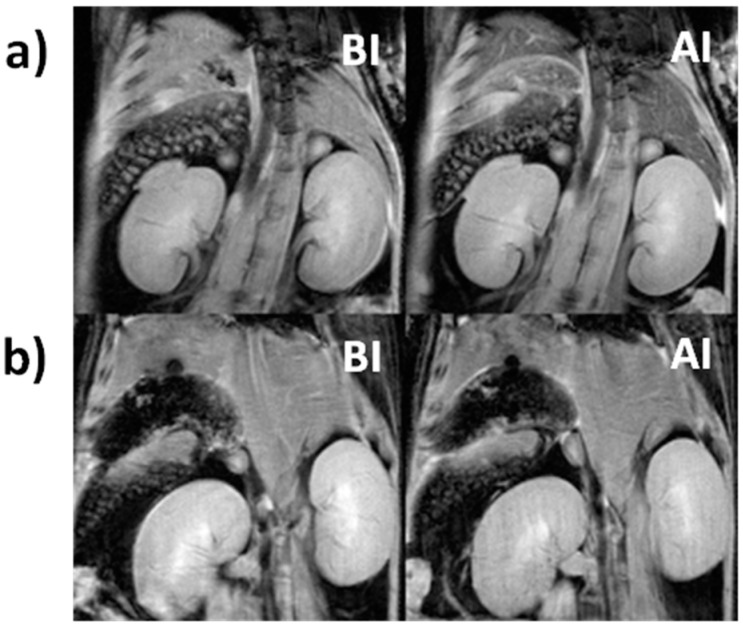
Coronal magnetic resonance (MR) images of the abdominal cavity of mice before (BI) and after (AI) injection of aged (**a**) or freshly (**b**) prepared solutions of IONPs.

During imaging sessions, a series of anatomical images were acquired before and after the injection of IONPs to properly allocate the organs of interest. Figure 4 shows a coronal view of the abdomen of a mouse, where the main organs of the RES, i.e., the liver (small fragment of it visible at the top of the images) and the spleen (the granular organ on the left side) are visible, together with the two kidneys (bean shaped organs). Images show the abdomen of mice at two different time points (before injection, BI, on the left and after injection, AI, on the right), into which a batch of aged IONPs (Figure 4a) and a batch of fresh IONPs (Figure 4b) were injected. At first sight, it could be visualized how the liver of the animal injected with aged solutions of IONPs darkened after injection, due to the accumulation of T2 contrast agent (IONPs).

**Figure 5:**
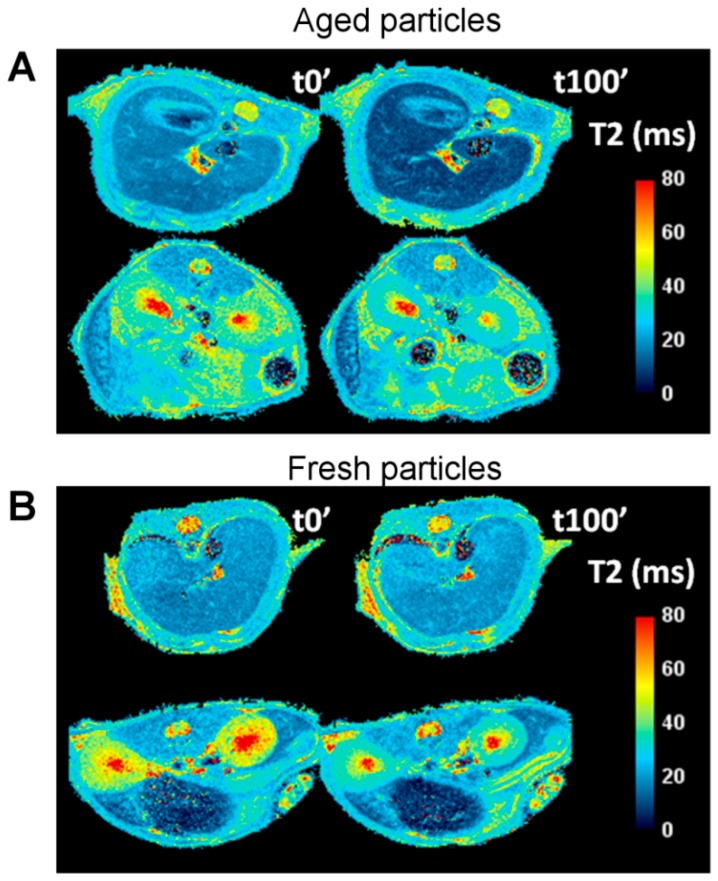
T2 relaxation maps at two different locations of the abdomen of a mouse showing the liver (top rows in both images) and the spleen and the kidneys (bottom row in both images). Two different time points are presented to reflect the changes in T2 values before (t0′) and 100 min after (t100′) the injection of the aged IONPs (**A**) and fresh IONPs (**B**).

In order to quantify the accumulation of nanoparticles in the different organs, transversal T2 parametric maps where constructed from acquired T2 multi-echo images (see methods section). Typical T2 maps of animals, pre- and post-injection of fresh and aged solutions, are presented in Figure 5, where the organs of the two principal routes of withdrawal of particles from bloodstream (RES and the urinary pathways) are visible.

Figure 5 is representative of the two different biodistribution patterns observed in our studies. For aged solutions of IONPs (Figure 5A) darkening of the liver and spleen indicate accumulation of IONPs in the RES organs (lowering T2 values). However, for a freshly prepared solution of nanoparticles (Figure 5B), no significant changes are observed at these two organs, while the kidneys show a clear change in relaxation times.

**Figure 6:**
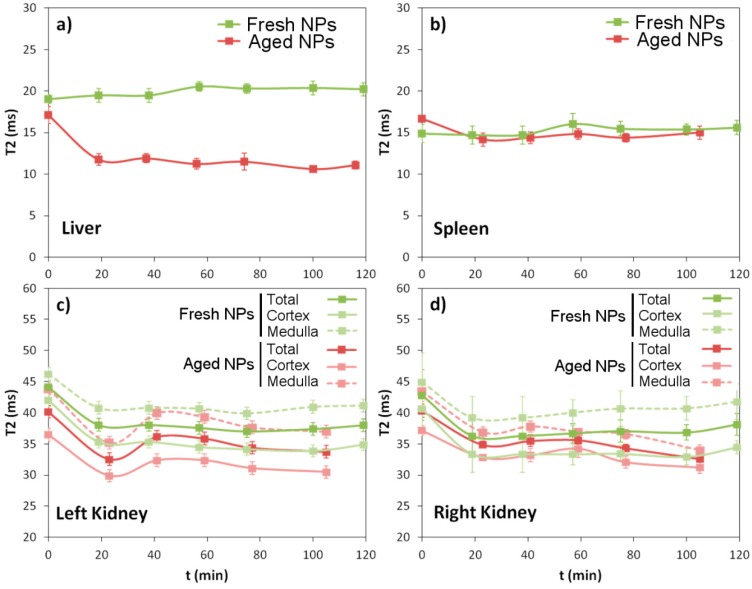
Evolution of the T2 relaxation times with time for the four different organs analyzed (liver (**a**), spleen (**b**), left kidney (**c**), and right kidney (**d**)), after the injection of the aged solution, (red) and a freshly prepared solution, (green) of IONPs in two representative animals of each group.

Studies were performed in a group of n = 6 (aged) and n = 7 (fresh) animals, and T2 values where measured in selected regions of interest (ROIs) for all organs, at t = 20 min interval for 120 min post injection of the contrast agent (giving a total experimental time of 3 h including pre-contrast imaging). ROI drawing was carefully performed to avoid the inclusion of regions of high T2 values that usually correspond to fluids like blood (portal vein and other blood vessels in the liver, and renal artery and/or vein in kidneys) or bile (gallbladder and bile ducts in the liver). In addition, two different analyses were performed for the kidneys, by selecting an ROI of the whole kidney, or two different ROIs, one corresponding to the medulla and the other to the cortex of the kidneys. Both kidneys were analyzed separately. Plots of T2 evolution with time for all these ROIs for a representative animal of each group are presented in Figure 6.

Figure 6 summarizes numerically what it is visually perceptible in Figure 5. When the solution of nanoparticles is used during the first few weeks after solution, they are able to avoid the RES and do not accumulate in the liver or spleen (green lines in Figure 6a,b), ending up in the kidneys, with preference to accumulate in their cortical region. It is noticeable that accumulation takes place already during the first 20 min, and no further reduction of T2 values is observed beyond this point. The drop in T2 values is higher than 20% in the kidneys. Conversely, when IONPs are injected after remaining circa 9 months in solution, a considerable drop in T2 values is observed in the RES organs (particularly in liver) in detriment of the kidneys, pointing to the loss of stealth capacity. Once more, nanoparticle accumulation seems to take place mainly during the first 20 min post injection, and T2 values only exhibit little changes after that point.

**Figure 7:**
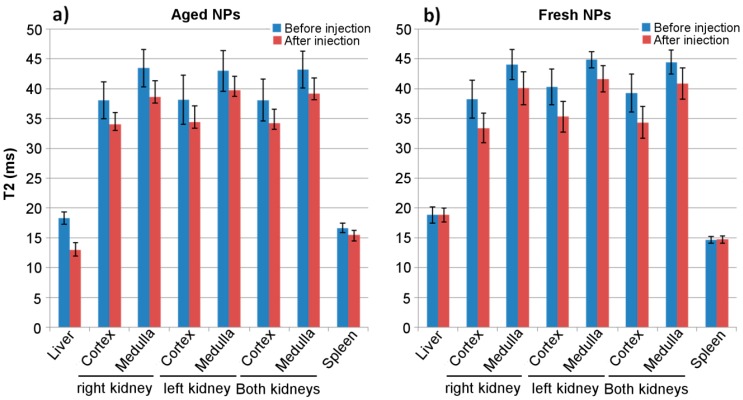
Mean (± SD) T2 relaxation values obtained for different organs before (blue) and after (red) injection of the aged (**a**) and fresh (**b**) nanoparticles.

Taking these results into account, a new plot was constructed to show the mean variation of relaxation times observed for each group (mean ± SD), and pooling together all the post contrast points for all animals, to present the mean change observed in T2 values, pre- and post-contrast, when fresh and aged nanoparticle solutions were used (Figure 7).

The value of T2 observed for the liver pre-contrast was very consistent for animals of both groups (aged, 18.3 ± 1.0 ms vs. fresh, 18.8 ± 1.4 ms) and with the post-contrast injection value of group treated with fresh nanoparticles (18.8 ± 1.2 ms), but descended 30% (to 12.9 ± 1.3 ms) after injection for the group treated with aged nanoparticles. The same behavior is observed for the spleen, with values that remain almost invariable for fresh nanoparticles (14.5 ± 0.6 ms pre- vs. 14.8 ± 0.6 ms post-contrast) but decreased by 10% for the aged ones (16.7 ± 0.8 ms vs. 15.5 ± 0.8 ms). For the kidneys, averaged mean drops of 10–13% of T2 values after injection have been observed for both groups. In global, these results point to the fact that, after 9 months in solution, nanoparticles have lost their stealth capacity, which is not the case for IONPs used within 3 weeks after solution.

These results evidenced the clear effects of NPs aging, seriously altering their biodistribution and availability, as reflected in the in vivo MRI experiments, despite maintaining similar physicochemical properties. We could demonstrate that the non-degraded or freshly prepared IONPs worked perfectly as negative contrast agents for the kidneys, where they mainly accumulated, inducing only minor effects in the liver and spleen, which confirms their stealth capacity and low retention by the RES. On the contrary, aging of IONPs in solution seems to induce some changes causing their fast uptake by the liver and spleen macrophages. Thus we further investigated the nature of these changes.

### 3.4. Stability of the Polymer Coating in Water: Hydrolysis of Ester Groups

**Figure 8:**
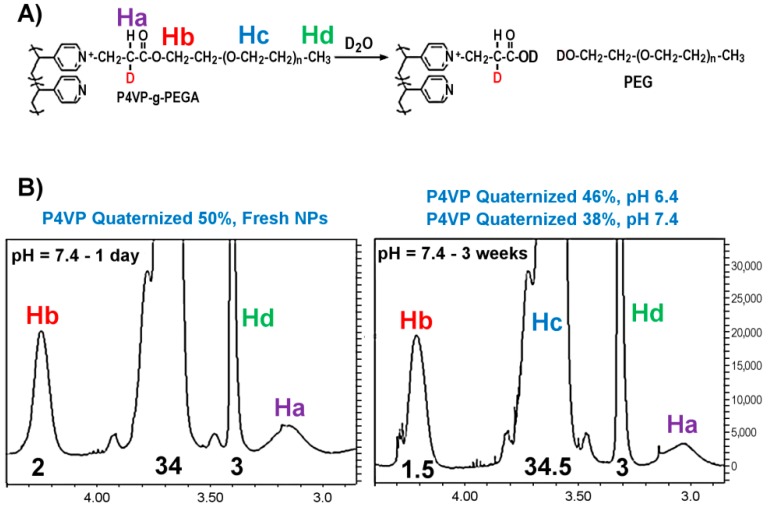
Time influence on the stability of the polymeric coating of PEG coated IONPs. Hydrolysis of ester groups present on the P4VP-g-PEG IONP coating at day 1 (**A**) and 3 weeks later (**B**).

Based on the results of the clear evidence of the different biodistributions for the differently aged IONPs, the stability of surface coating of the nanoparticles was studied with nuclear magnetic resonance (NMR) spectroscopy. Polymer coating was prepared in similar conditions than those used for the synthesis of the IONPs using methoxypolyethylene glycol acrylate and P4VP in deuterated water. The sample was stored at room temperature and analyzed periodically by NMR spectroscopy. We observed that there was a time dependent degree of hydrolysis of the ester groups present in the PEG coating of the IONPs (Figure 8A shows the hydrolysis and the Retro-Michael reaction and the signals of the protons that were analyzed with NMR).

The intensity of the signal of the terminal methoxy group (Hd) and the methylene of the ethylene oxide repeating units (Hc) were used as references, and compared with the intensity of the protons of the methylene group (Hb) attached to the acrylate group and the signal of proton (Ha) in the α position of the carbonyl group. After 3 weeks, the degree of quaternized pyridine in the coating decreased from 50% to 38%, which represents a 25% drop in grafting density when the pH of the sample was adjusted to pH 7.4. This loss is mainly due to the hydrolysis of the ester groups, although the appearance of a triplet next to Hb shows also the presence of small fraction of the starting monomer due to retro Michael reaction. Sample was extracted with dichloromethane and analyzed by mass spectroscopy (data not shown), settling the presence of the hydroxyl polyethylene glycol formed during the hydrolysis process. The degree of the hydrolysis was reduced just to 8% in the same period of time by adjusting the final pH of the sample to 6.4. Therefore, not only storage time, but also storage conditions (e.g., pH) have a key role in the preservation of the structural, and ultimately of the biomedical, properties of IONPs. These phenomena, along with other storing strategies, such as the influence of lyophilization-resuspension cycles, need to be explored in detail (as is taking place in our laboratories) to better understand how particles can be effectively preserved in time, for their translation to practical biomedical applications, performed far from synthetic laboratories (especially when GMP or similar grades of particles are required).

## 4. Conclusions

The use of nanomaterials, in particular IONPs, for biomedical applications is becoming very intense. The structure and physicochemical properties of these materials play a role of utmost importance in their biodistribution and availability. Several effective solutions have been proposed to increase circulation time of nanomaterials in the blood stream, avoidance of opsonization and the RES, the decoration of particles with PEG being among the most common ones. However, aging of particles in solution may affect the integrity of such coating, with dramatic consequences for the biomedical profile of the particles. This is a phenomenon that may require an in depth analysis, since regular inspection such as measurement of size, shape, or superficial charge may not be sufficient to detect such phenomenon. To take actions that preserve nanoparticles’ properties once synthesized is of paramount importance to ensure their potential biomedical properties are retained [33].
